# A quantitative metric for the comparative evaluation of optical clearing protocols for 3D multicellular spheroids

**DOI:** 10.1016/j.csbj.2021.01.040

**Published:** 2021-02-04

**Authors:** Akos Diosdi, Dominik Hirling, Maria Kovacs, Timea Toth, Maria Harmati, Krisztian Koos, Krisztina Buzas, Filippo Piccinini, Peter Horvath

**Affiliations:** a Synthetic and Systems Biology Unit, Biological Research Centre (BRC), H-6726 Szeged, Hungary; b Doctoral School of Biology, University of Szeged, H-6726 Szeged, Hungary; c Doctoral School of Computer Science, University of Szeged, H-6701 Szeged, Hungary; d Department of Immunology, Faculty of Medicine, Faculty of Science and Informatics, University of Szeged, H-6720 Szeged, Hungary; e IRCCS Istituto Romagnolo per lo Studio dei Tumori (IRST) “Dino Amadori”, Via Piero Maroncelli 40, I-47014 Meldola, FC, Italy; f Institute for Molecular Medicine Finland, University of Helsinki, FI-00014 Helsinki, Finland; g Single-Cell Technologies Ltd., H-6726 Szeged, Hungary

**Keywords:** Optical tissue clearing, Spheroid, Light-sheet microscopy, Focus metrics

## Abstract

3D multicellular *spheroids* quickly emerged as *in vitro* models because they represent the *in vivo* tumor environment better than standard 2D cell cultures. However, with current microscopy technologies, it is difficult to visualize individual cells in the deeper layers of 3D samples mainly because of limited light penetration and scattering. To overcome this problem several optical clearing methods have been proposed but defining the most appropriate clearing approach is an open issue due to the lack of a gold standard metric. Here, we propose a guideline for 3D light microscopy imaging to achieve single-cell resolution. The guideline includes a validation experiment focusing on five optical clearing protocols. We review and compare seven quality metrics which quantitatively characterize the imaging quality of spheroids. As a test environment, we have created and shared a large 3D dataset including approximately hundred fluorescently stained and optically cleared spheroids. Based on the results we introduce the use of a novel quality metric as a promising method to serve as a gold standard, applicable to compare optical clearing protocols, and decide on the most suitable one for a particular experiment.

## Graphical abstract

**Figure:**
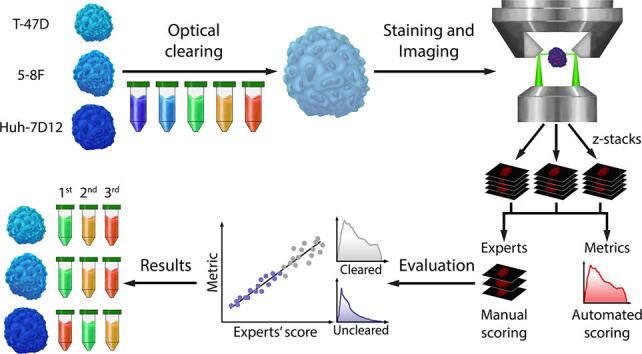

## 1 Introduction

Two dimensional (2D) monolayer cell cultures have been used extensively as model systems to evaluate the efficacy of compounds in drug discovery studies for decades. However, it has been demonstrated that culturing cells in 2D does not properly reflect the physiological properties of tissues and tumor-specific microenvironments [1]. As an attempt to find a more relevant model system for drug testing, three dimensional (3D) cell cultures have gained increasing attention [2]. Several 3D *in vitro* models are currently used in biological laboratories [3], the most common of which are spheroids where the cells are arranged into clusters in a sphere-like structure [4]. Spheroids mimic *in vivo* conditions and preserve the structure of the cells, making this model system remarkable for many biological research fields, such as drug discovery, tumor biology or immunotherapy [5], [6], [7]. Despite their advantages exploited in screening studies, large-scale image acquisition is still challenging in case of 3D samples. Single-cell phenotyping is one of the most promising drug screening approaches emerging nowadays [8], [9]. It is one of the most relevant techniques to monitor the morphological changes induced by pharmacological treatments in drug screening studies, allowing to understand the effects of the compounds tested. Light sheet-based fluorescence microscopy (LSFM) is widely used to visualize single cells composing the inner core of the spheroids [10]. LSFM obtains optical sections by moving the sample through a thin layer of laser light, also called a light sheet, which illuminates the focal plane of the detection path. Since LSFM provides high imaging speed with remarkably low photobleaching and high penetration depth [11], the technique is well suited for the imaging of large spheroids, typically up to 500 µm [12].

However, in spheroids, light scattering strongly limits imaging depth: scattering of both the excitation and emission lights results in a loss of fluorescence intensity and contrast. As a consequence, the imaging depth is restricted, practically allowing the screening of cells in the outer layer of the spheroids only. This light scattering effect is mainly explained by the discontinuities of the refractive index (RI) between and within spheroids [13]. To overcome this problem, many optical clearing protocols were established during the last decade [14]. Most of these methods aim to increase transparency of spheroids chemically, by equilibrating RI throughout the sample to reduce inhomogeneities in light scattering. To achieve this, various approaches have emerged, such as dehydration, solvent- and water-based techniques [13]. Although clearing protocols have been increasingly adopted for 3D cultures in cellular phenotyping assays [15], [16], the quantitative assessment of the efficiency of these methods is still challenging. Most of the studies focusing on newly developed clearing protocols used diverse qualitative and quantitative efficacy measures to assess the performance of the newly established clearing technique. However, due to the subjective aspects of human perception and the lack of gold standard metrics, many optical clearing protocols are available and used without a proper evaluation of their efficacy.

In this article, we report on developing and comparing novel metrics that measure the efficiency of optical clearing protocols for 3D images in an uniform way. We considered seven no-reference sharpness metrics for evaluating the clearing protocols and implemented those in a user-friendly open-source ImageJ/Fiji [17], [18] plugin named Spheroid Quality Measurement (SQM). To test their performance and usability, we created and shared a large 3D dataset [19] composed by 90 cancer spheroids and established a 3D analysis pipeline using five popular water-based clearing protocols, namely Clear^T^ [20] Clear^T2^ [20] CUBIC [19], [21], ScaleA2 [22] and Sucrose [19], [23]. We used the human experts’ evaluation of the 3D dataset as the ground truth and compared the correlation between the metrics and the human experts. We found that among the seven metrics, only intensity variance is suitable to quantitatively measure and evaluate different optical clearing protocols. Finally, we compared the efficiency of the clearing protocols on spheroids derived from three different human carcinoma cell lines with intensity variance metric and identified the best clearing protocols for each cell line. Based on these findings we support intensity variance as the gold standard metric to evaluate the efficacy of optical clearing protocols on 3D multicellular spheroids.

## 2 Results

### 2.1 3D pipeline

**Fig. 1:**
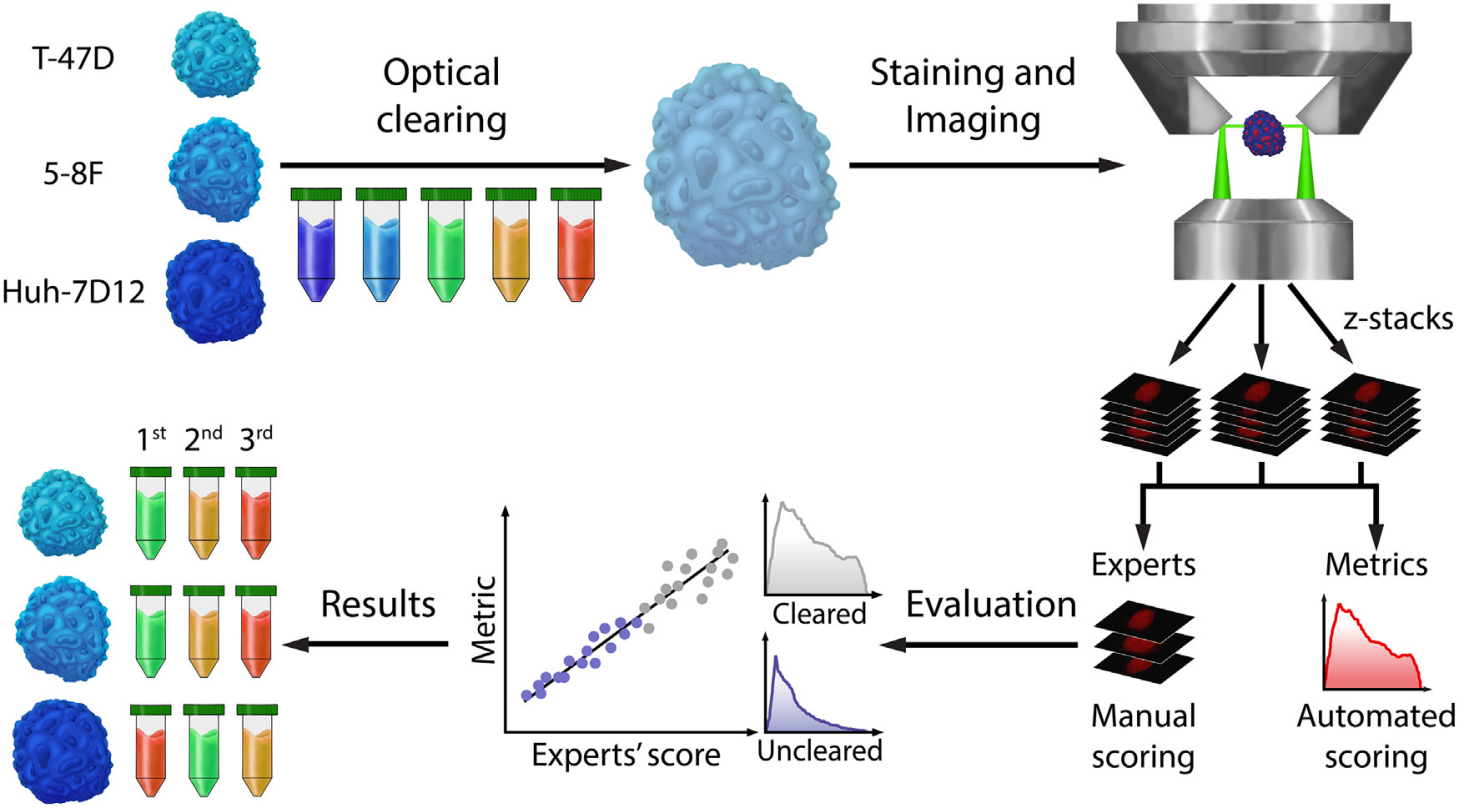
Representation of the 3D pipeline summarizing the concept of our experiments. Spheroids from T-47D, 5-8F, and Huh-7D12 cell lines with a similar size range (approx. 250 µm) were cleared with Clear^T^, Clear^T2^, CUBIC, ScaleA2, and Sucrose protocols, and the nuclei of the cells were labeled with DRAQ5 staining. For imaging, a Leica SP8 digital light-sheet microscope was used, yielding *z*-stack images. Ten experts evaluated the sharpness of the fluorescent images, and we compared their scoring with the tested quality metrics. Correlations between the quality metrics and human evaluation were calculated using Pearson’s correlation, and the metric with the best correlation was used to compare the efficacy of the optical clearing protocols applied on three types of spheroids originating from different cell lines.

**Fig. 2:**
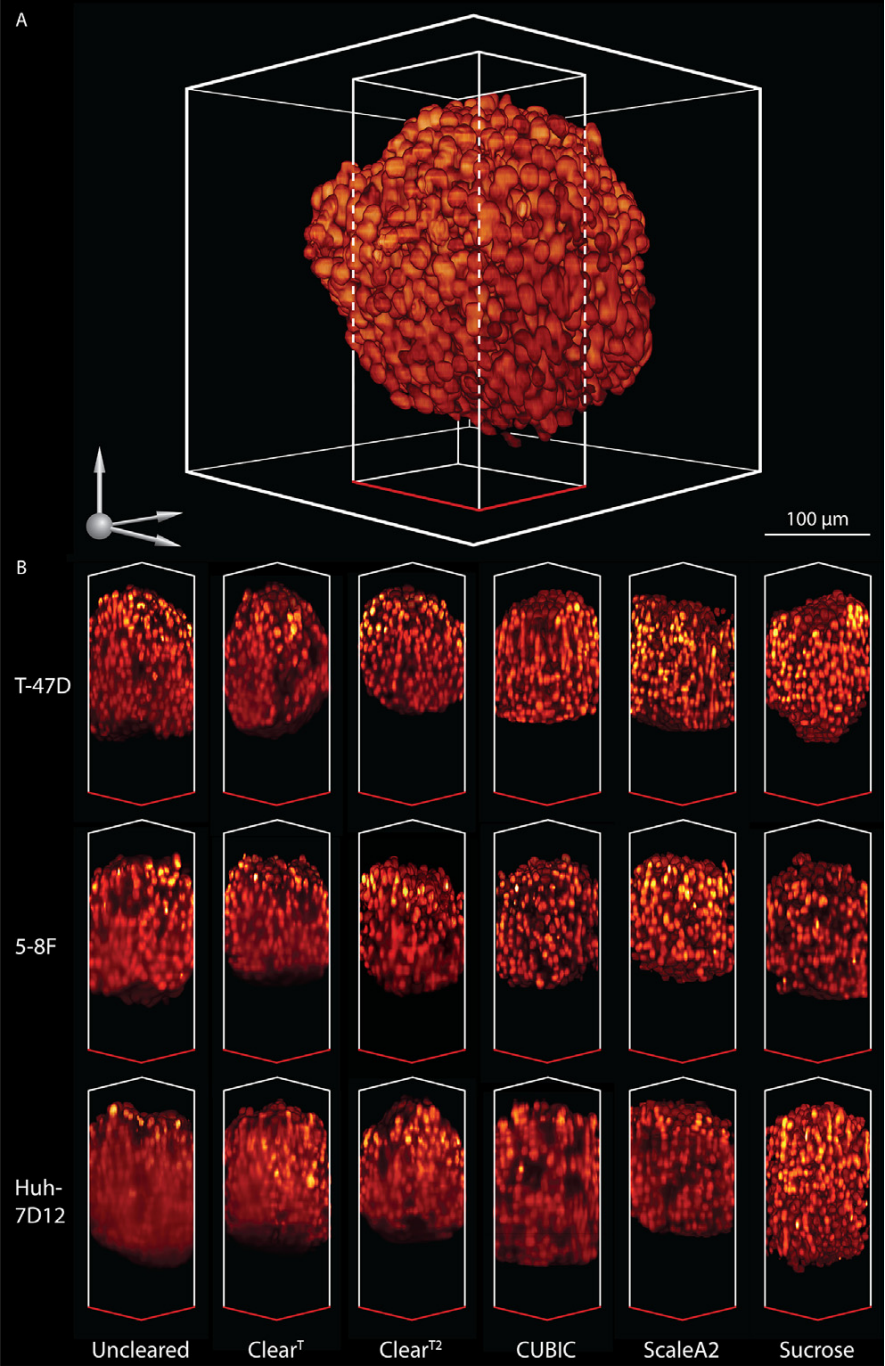
3D representation of the dataset. (A) 3D representation of a nuclei-labeled Huh-7D12 spheroid cleared with Sucrose optical clearing protocol. For visualization, a square (110 × 110 × 300 µm) from the middle part of the original spheroid was extracted. (B) For all the five clearing protocols, the same area was extracted from all the three cell lines. The scale bar represents 100 µm. The images were taken and visualized with a LAS X microscope software developed by Leica.

To evaluate the quality assessment metrics, we designed a 3D pipeline (Fig. 1). The seven metrics we consider here are commonly used to benchmark image sharpness in photos and videos, however, they characterize different aspects of the images. In order to validate these metrics, we first created a 3D dataset of cleared mono-culture spheroids (Fig. 2). All details are reported in [19]. Then, we asked ten microscopy experts (researchers that have been working with spheroid images and possess at least 5 years of experience in microscopy) to visually evaluate the sharpness of the images and we correlated their evaluations with the results of the seven metrics. Finally, to measure the efficacy of the clearing protocols, we used the metric that best correlated with the evaluation of the experts. In this article we report on our findings, organized as follows: Section 2.2 (i.e. **“Human evaluation”**) summarizes the results of the human evaluation of the 3D dataset. In Section 2.3 (i.e. **“Quantitative metrics”**) we discuss the results for image quality assessment using the seven objective no-reference sharpness metrics that we implemented as an ImageJ/Fiji plugin. The correlation between the quality metrics and human evaluation, as well as the performance of the 5 optical clearing protocols tested are presented in Section 2.4 (i.e. **“Correlation and clearing results”**).

### 2.2 Human evaluation

For a quantitative testing of the proposed metrics, a 3D light-sheet dataset of spheroids was used [19]. The dataset contained fluorescence stack images of 90 spheroids that included three cell lines (T-47D, 5-8F, and Huh-7D12), five clearing protocols (Clear^T^, Clear^T2^, CUBIC, ScaleA2, and Sucrose), and an uncleared group as a control (the number of spheroids was *n* = 5 for each group). Ten microscopy experts scored the LSFM images of the spheroids cleared with the optical clearing methods. The scores ranged from 1 to 5 (1 for the worst image quality and 5 for the sharpest image). To assess the consistency of each expert’s evaluation, some of the images were repeated. On average, the self-accuracy of the ten experts reached 81.6% for the evaluation of repeated images. The best-case consistency was 92.1% and the worst-case one was 74.5%, and only four of the experts recognized the repeated images during the evaluation process. In general, the experts were more uncertain in case of the highest scores (i.e. “good” – 4 or “very good” – 5) were considered to be appropriate. Therefore, the scores for the T-47D and the 5-8F spheroids were less consistent compared to the Huh-7D12 spheroid. According to the experts, they could differentiate between the top, the middle and the bottom regions of the spheroids. The average results for the evaluation executed by the ten experts are represented on a heatmap (Fig. S1**A)**. In general, the experts scored the T-47D spheroids higher, followed by the 5-8F spheroids, whereas the Huh-7D12 spheroids were characterized by the lowest scores. Comparing the optical clearing protocols, the results for Clear^T^ and Clear^T2^ were similar to the uncleared group, as the experts could hardly differentiate them from one another, however both of these clearing protocols decreased the size of the spheroids. Meanwhile, CUBIC, ScaleA2, and Sucrose protocols got higher scores for all the three regions of the spheroids, indicating that these optical clearings improved the transparency of the spheroids. We concluded that Sucrose was the only protocol that improved the image quality even at the bottom region for the Huh-7D12 spheroids, while the 5-8F and the T-47D spheroids had better scores when CUBIC and ScaleA2 protocols were applied. Both of these protocols reached very similar scores upon the experts’ evaluation, as no significant differences between these images could be detected by the human experts. Among the mentioned clearing protocols Sucrose resulted in minimal shrinkage, meanwhile CUBIC and ScaleA2 increased the volume of the spheroids. Regarding that currently there is no gold standard metric capable of assessing the differences between the different protocols, we considered the results for the experts’ evaluation as ground truth to compare the metrics for their appropriateness to quantify the performance of the clearing protocols tested.

### 2.3 Quantitative metrics

**Fig. 3:**
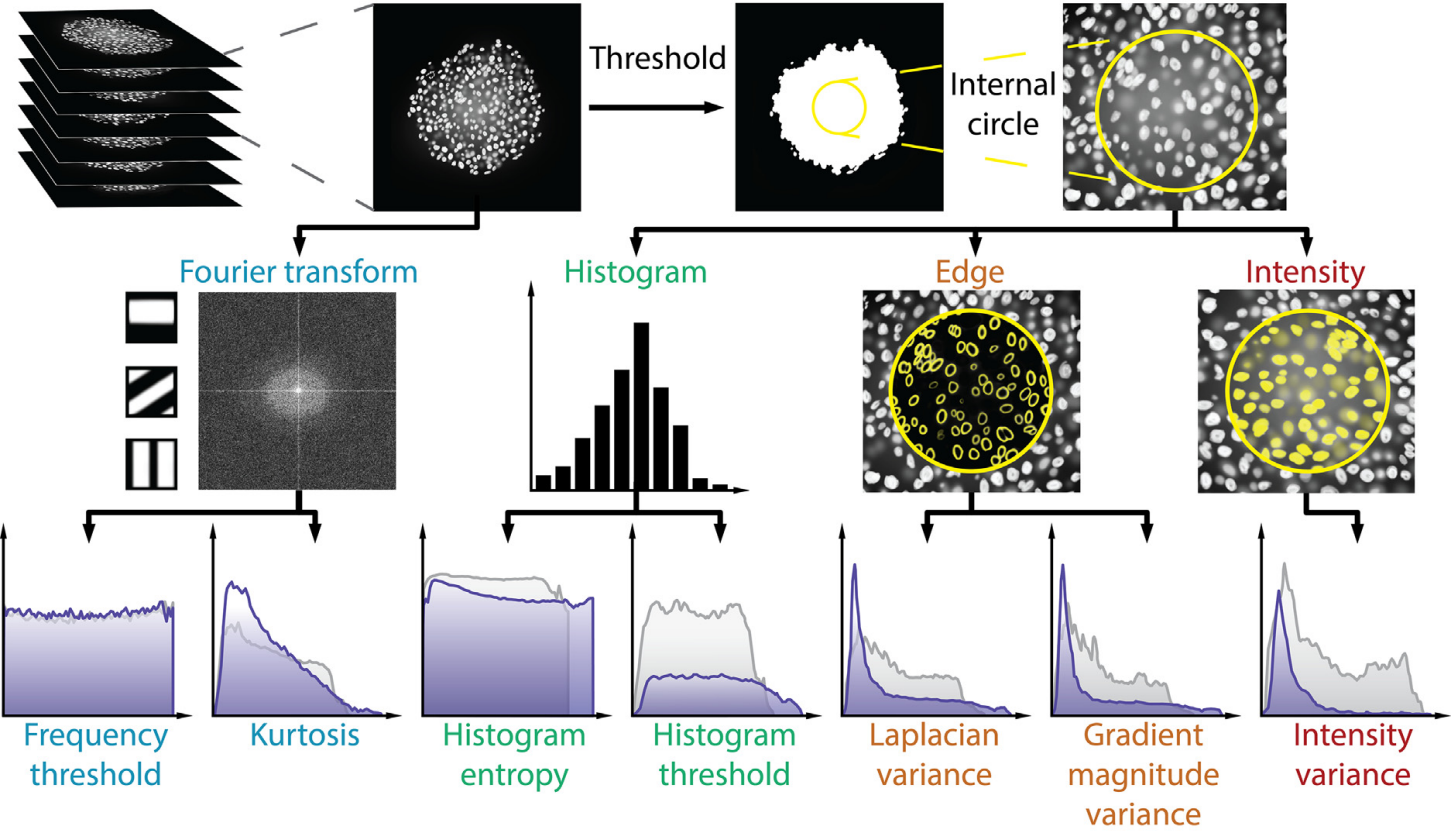
Conceptual figure for the workflow of the quantitative metrics. The metrics based on Fourier transformation were evaluated on the whole image, whereas for the other metrics the automatic Otsu threshold was applied. For histogram, edge and intensity based metrics, the internal circle option was also enabled to assess the information content at the center of the objects only. The results for each metric were visualized as a plot describing sharpness across the spheroid, where the coordinate in the *x*-axis represents the number of the image within the stack, and the *y*-axis is the score obtained by the metric. For histogram, edge and intensity based metrics, only the results for the internal circle are represented. Dark blue curves represent the uncleared spheroid, and gray curves represent the cleared spheroid. (For interpretation of the references to colour in this figure legend, the reader is referred to the web version of this article.)

We implemented seven metrics in a user-friendly ImageJ/Fiji plugin to assess the quality of microscopy images, namely intensity variance, Laplacian variance, gradient magnitude variance, histogram threshold, histogram entropy, kurtosis, and frequency threshold [24], [25], [26], [27], [28], and benchmarked them on 3D datasets. The metrics were applied on each optical section of the whole *z*-stacks independently, and the results were visualized. For histogram, gradient, and intensity based metrics, we enabled the threshold option to obtain information from the area of the spheroid. To handle the sharpness and contrast differences between the outer and inner layers of the spheroids, caused by the lateral light scattering effect, an internal circle option was also enabled which evaluates images inside the spheroids only. A schematic representation of the metrics is shown in Fig. 3, and an extended comparison of them is presented in Table 1. Detailed results for all the metrics evaluated on the Huh-7D12 cell line are shown in Fig. S2.

**Table 1:** Comparison of the metrics used in our experiments to quantitatively evaluate the imaging quality of spheroids. For the time comparison, 5 z-stacks with overall 295 images were processed using a consumer level laptop (Intel Core i5, CPU 1.80 GHz, 8 GB RAM).

Metric name	Value(s) used	Calculation of the metric	High value obtained	Low value obtained	Advantages	Drawbacks	Computational time (average)
Intensity variance	Grayscale pixel values	Variance of intensity values	There are many different intensities in the image which are far away from one another in brightness	There is just one intensity value in the image	General usability, strongly associated with image sharpness, fast	Needs fine-tuning in specific cases	0.82 s/image
Gradient magnitude variance	Magnitude of the partial image derivative vectors	Variance of gradient magnitudes	There are many edges in the image with various magnitude values	There are no edges in the image, the image is homogeneous	Strongly associated with image sharpness, widely used in autofocus detection, fast	Very sensitive to noise, yields high values for blurred images with very sharp regions	0.86 s/image
Laplacian variance	Second order image derivative values	Variance of the sum of second order derivatives	There are many edges in the image with various sharpness	There are no edges in the image, the image is homogeneous	Strongly associated with image sharpness, widely used in autofocus detection, fast	Noise sensitive, edges are not always indicative of image quality	0.92 s/image
Histogram thresholding	Image histogram values	Sum of histogram bin values above a predefined threshold (mean grayscale value), multiplied by their occurrence values	There are a lot of bright pixels in the image (theoretically when every pixel has the brightest value)	The pixels in the image have low brightness (theoretically when every pixel has 0 value)	Easy to calculate, fast	Applicable for image quality assessment in very specific cases only	0.88 s/image
Histogram entropy	Image histogram values	Shannon entropy of the histogram as a vector of the occurrence values	Every histogram bin has the same value	Only 1 histogram bin has all the values	Strong theoretical background, used for image quality assessment, fast	Not closely related to image sharpness, histogram information might be misleading	0.94 s/image
Frequency thresholding	FFT magnitude image intensities	Sum of the intensities in the FFT image with a high-pass filter	Image contains a lot of high frequency components (edges, sharp noise)	Image is mostly made of low frequency components (homogeneous objects)	Edges can be detected very effectively in the FFT image, which is closely related to sharpness	Noise sensitive, threshold value is important	0.35 s/image
Kurtosis	FFT magnitude image of the autocorrelation image	Kurtosis of the periodogram as a bivariate probability distribution	There are less high frequency components on the FFT of the autocorrelation image	There are more high frequency components on the FFT of the autocorrelation image	Theory suits well to sharpness assessment	Very slow, less intuitive than some other metrics	2.78 s/image

#### 2.3.1 Intensity variance

Intensity variance clearly differentiated the uncleared group from those that were cleared. The plot reaches the maximum variance in the top region of the spheroid, and constantly decreases towards the deeper layers. Higher steepness of the plot indicates that visibility inside the spheroid is limited. In general, the uncleared spheroids lost intensity variance from top to the center, mainly in the first outer third, while the cleared and more transparent spheroids retained higher values through their whole thickness (Fig. 3 and Fig. S2). The results for the internal circle yielded plots of similar shape, but separated the cleared spheroids better. This metric is one of the most basic methods, and it is a relatively fast approach for assessing image quality.

#### 2.3.2 Derivative based metrics

Gradient magnitude variance and Laplacian variance metrics are also pixel based. Metrics that use image derivatives require pixel operations in order to yield a transformed image on which the final assessments are executed. These derivative metrics provided plots similar to those yielded by the intensity variance method. Based on the evaluation of the whole spheroid, no differences between these metrics were detected, however, the results for internal circle assessment separated the uncleared and cleared groups, and changed the order of the clearing protocols (Fig. 3 and Fig. S2). These findings suggest that the internal circle option is suitable to compare the optical clearing protocols.

#### 2.3.3 Histogram based metrics

The histogram of a digital image shows the frequency of pixel intensities. Here we used two of the most popular histogram based methods for quality assessment, called histogram entropy and histogram thresholding. Analysing the entire spheroids, histogram threshold reached the maximum values at the middle and it separated clearing groups from each other (Fig. S2). While, histogram entropy metric yielded consistent plots and the differences between the clearing groups were hardly visible. Based on the evaluation of the internal circle, the difference between the uncleared and cleared spheroids were remarkable when using histogram thresholding metric, while histogram entropy plots showed the same consistent values across the spheroids. However, the assessment of the internal circle increased the differences and some of the cleared groups were separated from the uncleared group.

#### 2.3.4 Frequency based metrics

To investigate the frequency space, frequency threshold and kurtosis were implemented in SQM and were evaluated on the whole image, without internal circle option. Frequency threshold metric failed to visualize the differences between the cleared and uncleared spheroids. Overall, frequency threshold yielded consistent plots, and the different optical clearing protocols were characterized by similar curves (Fig. 3 and Fig. S2). On the other hand, the kurtosis metric distinguished the cleared groups from one another, and the shapes of the curves were similar to those yielded by the intensity and derivative based metrics. This finding suggests that the frequency space can provide additional information about the images. However, performing all the necessary calculations to get the bivariate kurtosis proved to be highly time consuming compared to the other metrics, and this drawback makes kurtosis less suitable for routine assessments.

### 2.4 Correlation and clearing results

#### 2.8.1 Results of the metrics

According to the experts’ evaluation, the Uncleared, Clear^T^ and Clear^T2^ image groups were characterized by lower scores, while CUBIC, ScaleA2, and Sucrose got higher scores (Fig. S1**A**). Accordingly, we expected a gap between the well-performing and worse performing clearing protocols. Frequency threshold metric was unable to differentiate between the clearing protocols (Fig. S2). Kurtosis metric, which can be calculated for the whole image only, yielded results similar to those obtained by intensity and edge based metrics, however, the slow processing time make this metric less effective. Histogram threshold metric distinguished between the optical clearing protocols, but the results did not match the ground truth: the ScaleA2 and the CUBIC protocols did not improve transparency compared to the uncleared group, which is in contrast to the results for the experts’ evaluation. We also measured the histogram threshold metric in the internal circle of the spheroids, where it showed greater differences between the cleared and uncleared groups, but the overall rank of the clearings was the same as for the whole spheroid. On the other hand, the histogram entropy metric, besides revealing differences among the clearing protocols, also matched the ground truth’s order of the protocols. Furthermore, the assessment of the internal circle separated the clearing groups revealing greater differences between the protocols. Intensity variance, gradient magnitude variance, and Laplacian variance metrics yielded plots with very similar shape and almost the same order of the clearing protocols. Furthermore, intensity variance metric yielded similar results for the whole spheroids and for the internal circle of the spheroids. Meanwhile, gradient magnitude and Laplacian metrics revealed greater differences between the clearings, based on the internal circle assessments. Histogram entropy, histogram threshold, and frequency threshold methods yielded different line charts. Besides from the different line charts, all the metrics were reckoned to be worthy of further investigation to compare their correlation with the experts’ evaluation.

#### 2.8.2 Correlation

To check if there is a quantitative metric able to reflect the experts’ evaluation, the Pearson’s correlation coefficient was computed between each metric and the ground truth by normalizing the results for all the spheroids. Here, we only discuss the intensity variance metric that showed the highest correlation with the experts either when applied to the whole spheroid or to the internal circle only. This makes it an optimal candidate to assess quality differences. Considerations for the other metrics are reported in Supp. Note 1, with a detailed version of all the correlations and the individual normalization depicted in Fig. S3.

**Fig. 4:**
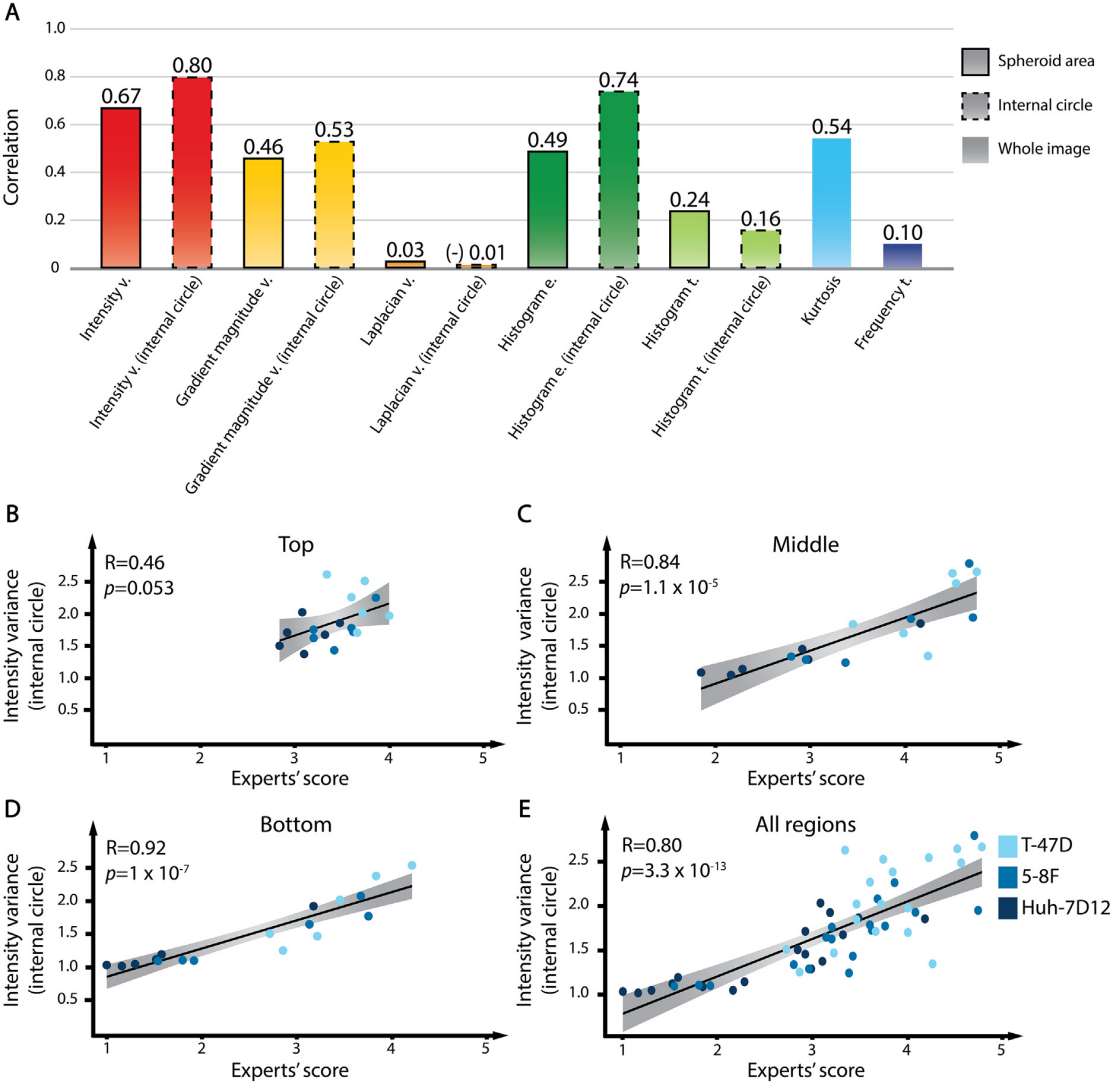
Correlation between the metrics and the experts’ evaluation. (A) Results for the Pearson’s correlation analysis between the metrics and the experts’ assessment. Metrics were evaluated considering all the cell lines together. Bounding boxes with dashed lines represent the results of the internal circle assessment. (B-D) Correlation results for the three regions of the spheroids. (E) For a reliable calculation of the correlation between a metric and the experts’ assessment (i.e. the ground truth), all three regions of spheroids were included. Dark-blue dots represent the Huh-7D12 spheroids; blue dots the 5-8F spheroids; light-blue dots the T-47D spheroids. The correlation was visualized with linear regression, and the Pearson’s correlation coefficient was calculated for all the spheroids. In total, 54 pairs were tested to assess the overall correlation between the metrics and expert assessment, whereas only 18 pairs were tested to demonstrate the correlations at the different regions. (For interpretation of the references to colour in this figure legend, the reader is referred to the web version of this article.)

The Pearson’s correlation coefficient for intensity variance resulted in 0.67 (Fig. 4**A**). The internal circle option improved the overall match with the experts’ evaluation and showed a higher correlation (0.80, Fig. 4**A and E**). The correlation between intensity variance and the experts’ scores was highest at the bottom regions of the spheroids and decreased at the middle and top regions. The Pearson’s correlation coefficient for the top, the middle, and the bottom regions were 0.46, 0.84, and 0.92 (Fig. 4**B-D**). Better image quality (like at the top region of the spheroids) and higher transparency of the spheroids weakened the correlations. This result might be contradictory, but it can be explained by the fact that the human decisions were less consistent with images of better quality because the experts found it difficult to decide the optimal score for them. In general, intensity variance metric showed the strongest correlation with the human scores in cases when the experts well distinguished the groups from one another (like at the bottom regions), independently of the type of the spheroids. Moreover, a stronger correlation between intensity variance and the experts’ scores was revealed for the Huh-7D12 spheroids compared to the T-47D and 5-8F spheroids. We reckon that the fairly good overall 0.80 Pearson’s coefficient for the correlation between intensity variance metric and the experts’ scores, indicates that intensity variance might be reliably used for the quality assessment of optical clearing protocols.

#### 2.8.3 Clearing efficacy based on the intensity variance metric (based on internal circle assessment)

**Fig. 5:**
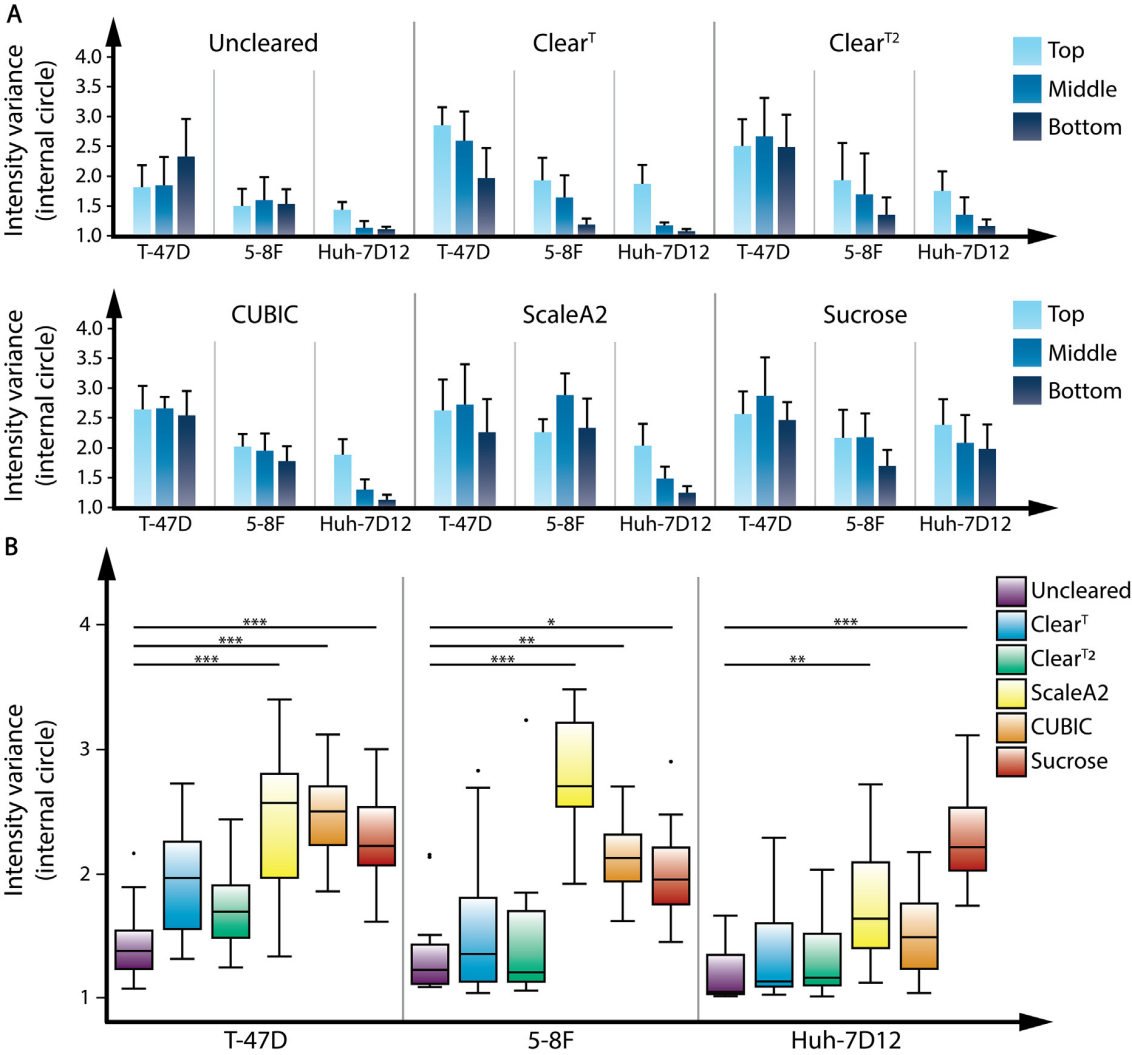
Results for the clearing protocols (A) Performance (efficacy) of the clearing protocols on the spheroid types derived from different cell lines. Three types of spheroids were compared at three regions (top, middle, bottom) using intensity variance metric with the internal circle option, after applying each clearing protocol. The scores represent the results for the different types of spheroids treated with the same clearing protocol. (B) Comparison of the clearing protocols on each spheroid type to reveal the most appropriate method for each cell line. Intensity variance was used with the internal circle option to assess the quality of each protocol. The efficacy of each clearing protocol was compared to the uncleared spheroids of the same type. Each cleared group contains five spheroids that were divided into three regions (top, middle and bottom), yielding 15 values per group for quality assessment. *p ≤ 0.05; **p ≤ 0.01; ***p ≤ 0.001.

The correlation analysis confirmed that intensity variance metric yielded the highest scores both for the whole spheroid and for the internal circle assessments. Thus, in our further experiments we used this metric to visualize the spheroids treated by the five optical clearing protocols tested. Our primary aim was to find the best optical clearing protocol which is capable of clearing all the three types of spheroids in an appropriate manner. The metrics do not require a similar efficacy between the protocols to be compared. However, comparing clearing methods with a similar efficacy it is easier. Regarding the methodology, it must be emphasized that the comparison of different clearing protocols presumes that every single protocol is applied on all different spheroid types, one by one, and the cleared spheroids are analyzed by the chosen metric to reveal how each protocol performs on the different cell lines (Fig. 5**A**). In contrast, when different clearing protocols are applied in parallel on a well-defined spheroid type, the results can only show the best protocol for the cell line of interest, but this type of experiment is inappropriate for the evaluation of the relative efficacy of the different clearing protocols on various spheroid types (Fig. 5**B**). This methodological issue is explained by the fact that different normalization scales are used for the assessment of each sample type (Fig. 5 and Fig. S4). All three types of spheroids were treated with each clearing protocol, and were analyzed using intensity variance metric, separated to top, middle and bottom regions. Using intensity variance, the T-47D spheroid reached the highest scores, followed by 5-8F and Huh-7D12, which confirmed the transparency differences among the spheroids derived from various cell lines. In all cases, the top and the middle regions of the spheroids reached higher scores than the bottom, which also confirms the presence of vertical light scattering. The results for internal circle assessments differed from the results related to the whole spheroid. Regarding the efficacy of the optical clearing protocols, the analysis revealed that Sucrose increased transparency for all the spheroids (Fig. 5**A**), but the total score for the three spheroid types were not similar in all the regions. The efficacy of the clearing protocols revealed that ScaleA2 and CUBIC clearing protocols were successful on certain cell lines only, but not on all the three types of spheroids (Fig. 5**B**). When the cleared groups were compared to the control group, we noticed that Clear^T^ and Clear^T2^ increased transparency especially at the top region of the spheroids. Although they both slightly improved the scores, the results did not differ significantly from the scoring of the uncleared spheroids (Fig. 5**A and B**). In case of the T-47D spheroids, CUBIC, ScaleA2, and Sucrose protocols improved transparency significantly, but the differences among these groups were minimal. In case of the 5-8F spheroids, ScaleA2 protocol significantly improved image quality, yielding high quality images with low background signals. For the Huh-7D12 spheroids, Sucrose reached the highest scores (Fig. 5**B**). In general, the scores for the whole spheroid assessments were higher than those for the internal circle assessments, because the outer parts of the spheroids improved the values (Fig. S4). Due to the lateral illumination by the light-sheet microscope, the higher contrast of the nuclei at the outer shell reduced the accuracy of the comparison, leading to modified overall results. Based on the whole spheroid assessments, all clearing protocols improved the overall scores for each cell line. The differences among CUBIC, ScaleA2 and Sucrose clearing protocols decreased, but the order of the protocols slightly changed in case of the 5-8F and Huh-7D12 spheroids (Fig. S4**B**).

## 3 Discussion

Regarding microscopy image analysis of 3D *in vitro* models, the quality of the acquired images depends on the size, shape and incubation time and cell line composing the 3D model. These features and conditions often lead to inconsistency in the acquired images’ quality. The efficacy of optical clearing protocols applied to enhance image quality highly depends on the cell line. Thus, choosing the best optical clearing protocol for the target 3D model is crucial. Currently, plenty of different approaches are utilized to assess the efficiency of clearing protocols for 3D samples. However, there is no gold standard metric to quantitatively evaluate and compare the different clearing protocols. In most studies of newly developed clearing protocols both qualitative and quantitative approaches were used to assess clearing efficacy. Among the qualitative approaches, most of the researchers simply used subjective scoring, based on microscopy images before and after optical clearing. These experiments are based on the ratings of brightfield and fluorescence microscopy images [29]. In fact, this method is appropriate when we simply have to decide whether fluorescent signals are present or absent after the clearing process, or when we simply need to distinguish different clearing protocols based on brightfield images. However, these measurements are time-consuming, and are not feasible when hundreds of 3D images are to be compared.

Some of the qualitative metrics aim to assess the changes in various fluorescence intensity profiles: intensity/contrast increases in both lateral and axial dimensions; tissue transparency improves due to clearing. The basic concept of this approach is the signal-to-background (SNB) ratio, which is defined as the ratio between the mean signal and the standard deviation of the background signal in the intensity profile. To obtain this ratio, an intensity threshold is usually applied to determine background (values below threshold) and signal intensities (values above threshold) [30]. As another option, calculating the changes in mean fluorescence intensity at different imaging depths of a 3D sample can also be used to assess lateral or axial imaging depth [31], [32]. Furthermore, corrected total cell fluorescence (CTCF) was also introduced to monitor the loss of the fluorescent signal in response to optical clearing [31], [32], [33], [34]. A recent study has developed an improved signal-to-noise ratio (SNR) based method to characterize depth-dependent signal intensity [35]. Regarding the well-established correlation between image quality improvement and fluorescence intensity changes, assessing these alterations is quite a popular approach in literature. However, as a disadvantage of intensity values, the results are highly sensitive to staining quality and imaging settings, such as exposure time. Furthermore, the results of these analyses are highly dependent on the applied threshold as well, which might easily lead to the misinterpretation of total intensity changes for a whole spheroid.

A different approach to assess the performance of an optical clearing protocol is based on the concept that the number of well-segmented nuclei should be increased within the entire spheroid as image quality improves [36]. An obvious drawback of using the segmentation-based approach (to compare the efficacy of various clearing protocols) is that the results are highly dependent on the precision of the applied segmentation approach.

Based on these considerations, instead of using only the intensity values of the images or the information for the segmented nuclei, we tested seven metrics which are used to characterize blurriness of general photos and videos. Implemented in a user-friendly ImageJ/Fiji plugin, the seven metrics were tested on a large public available 3D dataset [19], quantifying the quality of microscopy images of different spheroid types. The results for these metrics-based analyses were compared to the sharpness ratings of the images executed by ten human experts, and the correlation between the two approaches was assessed. Among the seven metrics tested, intensity variance obtained the highest correlation with the experts’ evaluation. In this case, the results for the whole spheroid and for the internal circle showed relatively strong correlations with ground truth. Comparing the results for the different regions (top, middle, bottom) of the spheroid, we found that this metric strongly correlated with ground truth at the bottom and middle regions, while the correlation was only acceptable in the top region. The correlation between intensity variance metric and the human scores was the strongest in those cases when the experts could consistently differentiate the groups from one another, like in case of images of the bottom and middle regions. Meanwhile, for images of similar quality, such as images of the T-47D and 5-8F spheroids or images of the top regions, the experts could not distinguish the different clearings protocols. Since, the experts’ evaluation was the least consistent with the T-47D and 5-8F spheroids, we have concluded that even the best metric may not definitely show a really strong correlation with human assessment in these cases. However, an overall good correlation (Pearson 0.80) between the metrics and the experts suggests that intensity variance metric in point may serve as a quantitative tool to evaluate the relative efficacy of optical clearing protocols. Our results indicated that the appropriate protocol for optical clearing strongly depends on the cell line composing the spheroid. Furthermore, by comparing the results for the whole spheroid and for the internal part separately, we were able to measure the lateral light scattering effect. This effect is common with light-sheet microscopy systems where the samples are illuminated from the side resulting in great differences between the outer region and the internal parts of the spheroid. The results for the internal circle showed higher correlation with the experts’ evaluation, suggesting that image analysis data related to the internal part of the spheroid could be applicable in practice.

As expected, none of the tested optical clearing protocols performed equally well on all three cell lines. In case of the T-47D cell line, which forms the least compact spheroids, Sucrose, CUBIC and ScaleA2 protocols proved to be equally effective, yielding no significant quality differences among the cleared groups. The 5-8F spheroids showed the best image quality with the ScaleA2 clearing protocol. Finally, regarding the Huh-7D12, only Sucrose clearing protocol was able to visualise single nuclei located at the bottom regions of the spheroids. We also tested the reversible Clear^T^ and Clear^T2^ protocols and found that in case of the Clear^T^ protocol image analysis improved after the spheroids were washed, which suggests that this clearing method is in fact not completely reversible. Also, we could measure the differences between Clear^T^ and Clear^T2^ precisely, which provides a practical benefit to distinguish between slightly different clearings. Specifically, as these two protocols differ in a single component only, the effects of a protocol’s composition on spheroid transparency can also be evaluated based on image analysis.

In summary, we introduced seven metrics for image quality assessment, implemented in SQM, a user-friendly open-source ImageJ/Fiji plugin. We aimed to find a metric suitable for the quantitative assessment of *z*-stack images of 3D spheroids, allowing to compare optical clearing protocols without pre-processing of images. We tested the correlation between the metrics and the human experts’ evaluation (regarded as the ground truth), and found that of the seven implemented metrics, only intensity variance showed a good correlation, at least in the bottom and middle regions, with the experts’ assessment. This metric is suitable to quantitatively compare different optical clearing protocols and spheroids derived from various human carcinoma cell lines. Based on these findings, we support intensity variance as the gold standard metric to quantitatively compare optical clearing protocols.

## 4 Materials and methods

### 4.1 Intensity variance

For the intensity metric [25] the average and variance are calculated with the following formulas:

(1) $$I_{\mathrm{average}}=\frac{1}{\mathrm{MN}}\sum_{i=1}^{N}\sum_{j=1}^{M}I\left(i,j\right),$$

(2) $$\mathrm{Var}=\frac{1}{\mathrm{MN}}\sum_{i=1}^{N}\sum_{j=1}^{M}{\left(I\left(i,j\right)-I_{\mathrm{average}}\right)}^{2},$$

where I is the image with a resolution of M×N. Images with high variance indicate that there are pixels from very dark to bright values, which may suggest that the image is not blurred.

### 4.2 Derivative based metrics

Image gradient calculation is based on the differentiation of multivariable functions [25]: the partial derivatives of the image function $I\left(x,y\right)\left(\nabla I\left(x,y\right)\;=\;{\left(\frac{\partial I}{\partial x},\frac{\partial I}{\partial y}\right)}^{T}\right)$ represent the sharpness of the horizontal and vertical edges in the original image. This can be visualized by the magnitude of the gradients ($\left|\nabla I\left(x,y\right)\right|=\sqrt{{\left(\frac{\partial I}{\partial x}\right)}^{2}+{\left(\frac{\partial I}{\partial y}\right)}^{2}}$). After that, the mean and the variance of the gradient image can be calculated. Our implementation contains the variance of the gradient magnitude values:

(3) $$G=\frac{1}{\mathrm{MN}}\sum_{i=1}^{N}\sum_{j=1}^{M}{\left(|\nabla I\left(i,j\right)\left|-\right|\nabla {I|}_{\mathrm{average}}\right)}^{2},$$

where

(4) $${\left|\nabla I\right|}_{\mathrm{average}}=\frac{1}{\mathrm{MN}}\sum_{i=1}^{N}\sum_{j=1}^{M}\left|\nabla I\left(i,j\right)\right|.$$

Second order derivatives can also be used for focus detection, for example with the Laplacian operator [26]. The Laplacian of an image can be calculated with the following formula:

(5) $$L\left(x,y\right)=\frac{\partial^{2}I}{\partial x^{2}}+\frac{\partial^{2}I}{\partial y^{2}}.$$

Next, we calculate the variance of the Laplacian the same way as we did for the gradient magnitudes.

### 4.3 Histogram based metrics

The approach defines a threshold value T, and sums brightness values above that threshold, weighted by the number of pixels with that particular intensity. T is usually chosen as the average intensity in the image. Histogram threshold [27] metric is calculated with the following equation:

(6) $$M_{\mathrm{hist}}=\sum_{i|x_{i}>T}x_{i}f\left(x_{i}\right),$$

where $x_{i}$ are the pixel intensities and f is the histogram function.

Another histogram metric can be calculated using entropy [27], which is more precise for image quality assessment. The entropy is higher when the intensities are less predictable, i.e. they are more varied and not homogeneous. It is calculated as follows:

(7) $$M_{\mathrm{ent}}=-\sum_{i}\left(\frac{f\left(x_{i}\right)}{H_{\max}}log_{2}\frac{f\left(x_{i}\right)}{H_{\max}}\right),$$

where $H_{\max}$ is the vertical maximum of the histogram.

### 4.4 Frequency based metrics

Frequency based metrics rely on the 2 dimensional Fourier transformation, which converts an image from its original space to frequency space. It breaks down the image to a sum of weighted sine and cosine waves. Each pixel intensity at a position (u,v) represents the amplitude of the function with a certain frequency component characterized by u and v. Around the center of the resulting image lower frequency components are found, so a high amplitude in the middle corresponds to a homogeneous territory in the original image. With a growing distance from the center alongside concentric circles, higher frequency components are found. High amplitudes at the sides of the Fourier image usually correspond to sharp edges or noise in the original image.

The discrete Fourier transformation of an image can be formalized as follows:

(8) $$F\left(u,v\right)=\sum_{m=-\infty }^{\infty }\sum_{n=-\infty }^{\infty }I\left(m,n\right)e^{-2\pi i(um+vn)}.$$

The calculated function $F\left(u,v\right)$ is a complex function, so for visualization, $|F\left(u,v\right)|$ is typically used:

(9) $$\left|F\left(u,v\right)\right|=\sqrt{{Re\left(F\left(u,v\right)\right)}^{2}+{Im\left(F\left(u,v\right)\right)}^{2}},$$

where Re(z) and Im(z) are the real and imaginary parts of a complex number z.

One metric that can be calculated in the Fourier space is the frequency threshold metric [25]. Exactly how to measure sharpness in this domain varies. In this study, we defined a frequency threshold, and summed the amplitude values above that value only. This means that we sum the pixel values in the Fourier space outside a circle mask with radius r. Applying the mask is equivalent to multiplying the Fourier transformed image by a high-pass filter:

$$\mathrm{mas}k_{1}:=\left\{\left(i,j\right)|j^{2}+i^{2}>r^{2}\right\},$$

(10) $$M_{\mathrm{freq}}=\sum_{\left(i,j\right)\in mask_{1}}\left|F\left(i,j\right)\right|.$$

Kurtosis was proposed as an image sharpness measure [28]. The frequency space is utilized here as well, however, instead of using the Fourier magnitude image, kurtosis metric is calculated on the periodogram of the image, which is the Fourier transform of the autocorrelation image. The periodogram can be calculated as

(11) $$A\left(u,v\right)=\left|F\left(u,v\right)*\overset{-}{F}\left(u,v\right)\right|$$

where $\overset{-}{F}\left(u,v\right)$ is the complex conjugate of $F\left(u,v\right)$. To calculate the kurtosis of the periodogram, it is beneficial to regard it as a probability distribution. This can be achieved by normalization. We denote the frequencies at certain points of the periodogram by $u_{i},v_{j}\left(i=1,2,\cdots ,N\right)$, and calculate the normalized image with

(12) $$h\left(u_{i},v_{j}\right)=\frac{A\left(u_{i},v_{j}\right)}{\sum_{n}\sum_{m}A\left(u_{n},v_{m}\right)}$$

for every i,j=1,2,⋯,N.

The bivariate kurtosis of our probability distribution is calculated as

(13) $$\beta_{2,2}=\frac{\gamma_{4,0}+\gamma_{0,4}+2\gamma_{2,2}+4\rho_{12}\left(\rho_{12}\gamma_{2,2}-\gamma_{1,3}-\gamma_{3,1}\right)}{{\left(1-\rho_{12}^{2}\right)}^{2}},$$

where

(14) $$\gamma_{k,l}=\frac{\sum_{i=1}^{N}\sum_{j=1}^{N}h\left(u_{i},v_{j}\right){\left(u_{i}-\mu_{u}\right)}^{k}{\left(v_{j}-\mu_{v}\right)}^{l}}{\sigma_{u}^{k}\sigma_{v}^{l}},$$

and $\rho_{12}$ is the normalized correlation between u and v ($\rho_{12}=\frac{\sigma_{\mathrm{uv}}}{\sigma_{u}\sigma_{v}}$). The marginal means and variances of $h\left(u,v\right)$ are

(15) $$\mu_{u}=\sum_{i=1}^{N}u_{i}\sum_{j=1}^{N}h\left(u_{i},v_{j}\right),\mu_{v}=\sum_{j=1}^{N}v_{j}\sum_{i=1}^{N}h\left(u_{i},v_{j}\right),$$

and

(16) $$\sigma_{u}^{2}=\sum_{i=1}^{N}{{(u}_{i}-\mu_{u})}^{2}\sum_{j=1}^{N}h\left(u_{i},v_{j}\right),\sigma_{v}^{2}=\sum_{j=1}^{N}{{(v}_{j}-\mu_{v})}^{2}\sum_{i=1}^{N}h\left(u_{i},v_{j}\right).$$

Contrary to other metrics, a low number for kurtosis indicates a well-focused image, whereas high metric values suggest that the measured image is blurry.

### 4.5 Variance of normalized values inside the spheroid

We posed some conditions for the assessments of the fluorescence images:

1. In an image of a spheroid, our volume of interest is the spheroid itself only, the background contains no relevant information, so we do not want to use it.
2. Intensity values highly depend on the quality of the staining and image acquisition. Metrics can behave differently for higher and lower intensity ranges, therefore, normalization is necessary.
3. Certain clearing agents are not appropriate to penetrate into the spheroids, as a result the edges become very sharp, while the internal part remains blurred. For most of the metrics, this can result in an overall high contrast value due to the very sharp edges on the side, which may lead to misinterpretation. Therefore, we assess both the whole spheroid and the internal part of the spheroid, and compare the results.

To satisfy the 1st criterium, we applied a threshold to the spheroids, which can easily be achieved by the Otsu threshold algorithm: the spheroid area is very well distinguishable from the background based on the image histogram.

For intensity normalization (2nd criterium), we took the maximum intensity value of the 3D image stack and divided every pixel value with that.

To assess the internal part of the spheroid separately (3rd criterium), we calculated the center of the spheroid mass, slice by slice, using image moments:

$$\left(center_{x},center_{y}\right)=\left(\frac{M_{1,0}}{M_{0,0}},\frac{M_{0,1}}{M_{0,0}}\right),$$

where

(17) $$M_{i,j}=\sum_{x}\sum_{y}x^{i}y^{j}I\left(x,y\right).$$

We then calculated a desired pixel-based metric inside a circle with a radius of r and with the center calculated above:

$$\mathrm{mas}k_{2}:=\left\{\left(i,j\right)|{\left(j-center_{x}\right)}^{2}+{\left(i-center_{y}\right)}^{2}<r^{2}\right\}.$$

Our internal circle results were obtained with a 200 pixel radius circle. The usage of this is optional, and the radius can be changed. The choice of 200 pixel radius (~55 µm diameter) was to measure a large enough area (~10 nuclei at minimum) to have stable analysis but small enough to have reasonable coverage at the top and bottom of the spheroids.

### 4.6 Score calculation

We have introduced a metric which can be calculated after plotting the slice-by-slice metric plots. These steps were necessary to make the metrics comparable with the results of the expert evaluations. Let there be k different spheroid assessments, and let $g_{i}$ be the calculated metric plot for the i-th spheroid in the domain {1,⋯,n}, where n denotes the number of slices in the image stack, and let α be the maximum, and β be the minimum value from all the assessed $g_{i}$ plots:

$$\alpha =maxg_{i}\left(x\right),$$

$$\beta =ming_{i}\left(x\right),i=1,\cdots ,k.$$

We define our single-value score for every assessment with:

(18) $${\mathrm{score}}_{i}=\frac{1}{(\alpha -\beta )n}\sum_{j=1}^{n}{(g}_{i}\left(j\right)-\beta )$$

With this number, ranging between 0 and 1, the quality of spheroid images relative to one another can be characterized in a given assessment set. The theoretical maximum (1) is a perfect rectangle with a height of α-β, which would mean that an image with such a metric plot maintains the highest metric assessment across all of its slices. The theoretical minimum (0) is the constant zero function, which would mean that an image with such a metric plot gets a score of 0 for all of its slices. If an image maintains high metric values for most of the slices, its score will be closer to 1, whereas those images that consistently give low metric values, or the ones that have high values at the beginning, but then drastically decrease, will be closer to 0. In order to scale these scores to the human scoring system, we normalized all resulting values between 1 and 5. For all the metrics, the plots were divided into three equal regions, and the score calculation method described above was used. These steps do not change the results of the metrics, just scale them to match with the scoring system of the experts. For all five clearing protocol groups, as well as for the control group, the top, the middle, and the bottom regions of the spheroids were evaluated. Ten experts evaluated five spheroids from each clearing protocol group, but only one image from each region. The average of their scores was compared with the metrics. The results of the metrics were calculated almost the same way, except that the metrics evaluated all the images from each region, and the average scores were used for comparison. The whole spheroid and the internal circle were evaluated the same way, and the results were also calculated the same way. Next, the experts’ evaluation and the metrics’ scores were matched, and a linear regression analysis was carried out. For comparison, Pearson’s correlation was used.

All the metrics were implemented in a user-friendly open-source ImageJ/Fiji [17], [18] plugin named SQM. The results for score calculation are available as a csv file, saved by the plugin at the end of each image analysis process. SQM is implemented in Java and it works under Macintosh, Linux, and Windows 64-bit systems. There are no special hardware requirements. SQM can be downloaded from the Fiji plugin store, but it is also available at: https://bitbucket.org/biomag/qualitymetricplugin/downloads/

### 4.7 Statistical analyses

Statistical analyses were performed using the R software. The Kolmogorov-Smirnov test was utilized to check for normal distribution. For the statistical analysis of the optical clearing results, non-parametric Kruskal-Wallis test with Dunn’s multiple comparisons was performed. Significance level was set to α = 0.05 with a 95% confidence interval, and *p*-values were adjusted to account for multiple comparisons.

## Declaration of Competing Interest

The authors declare that they have no known competing financial interests or personal relationships that could have appeared to influence the work reported in this paper.
