# Supplementary material for: A quantitative metric for the comparative evaluation of optical clearing protocols for 3D multicellular spheroids
Source: Comput Struct Biotechnol J. 2021 Feb 4;19:1233–43. doi: 10.1016/j.csbj.2021.01.040 (PMC7907228; doi:10.1016/j.csbj.2021.01.040)
Supplement: Supplementary data 1 [file mmc1.docx]

**Supplementary Materials**

**Fig. S1 Heatmap representations of the dataset.**

**Fig. S2 Summary figure of the metrics on the Huh-7D12 spheroids.**

**Fig. S3** **Cell line-based correlation between metrics and experts’ evaluation.
Fig. S4 Comparison of the effects of 5 optical clearing protocols on spheroids, using intensity variance metric on the whole spheroid.**

**Supp. Note 1 Correlation results for all the metrics**

**
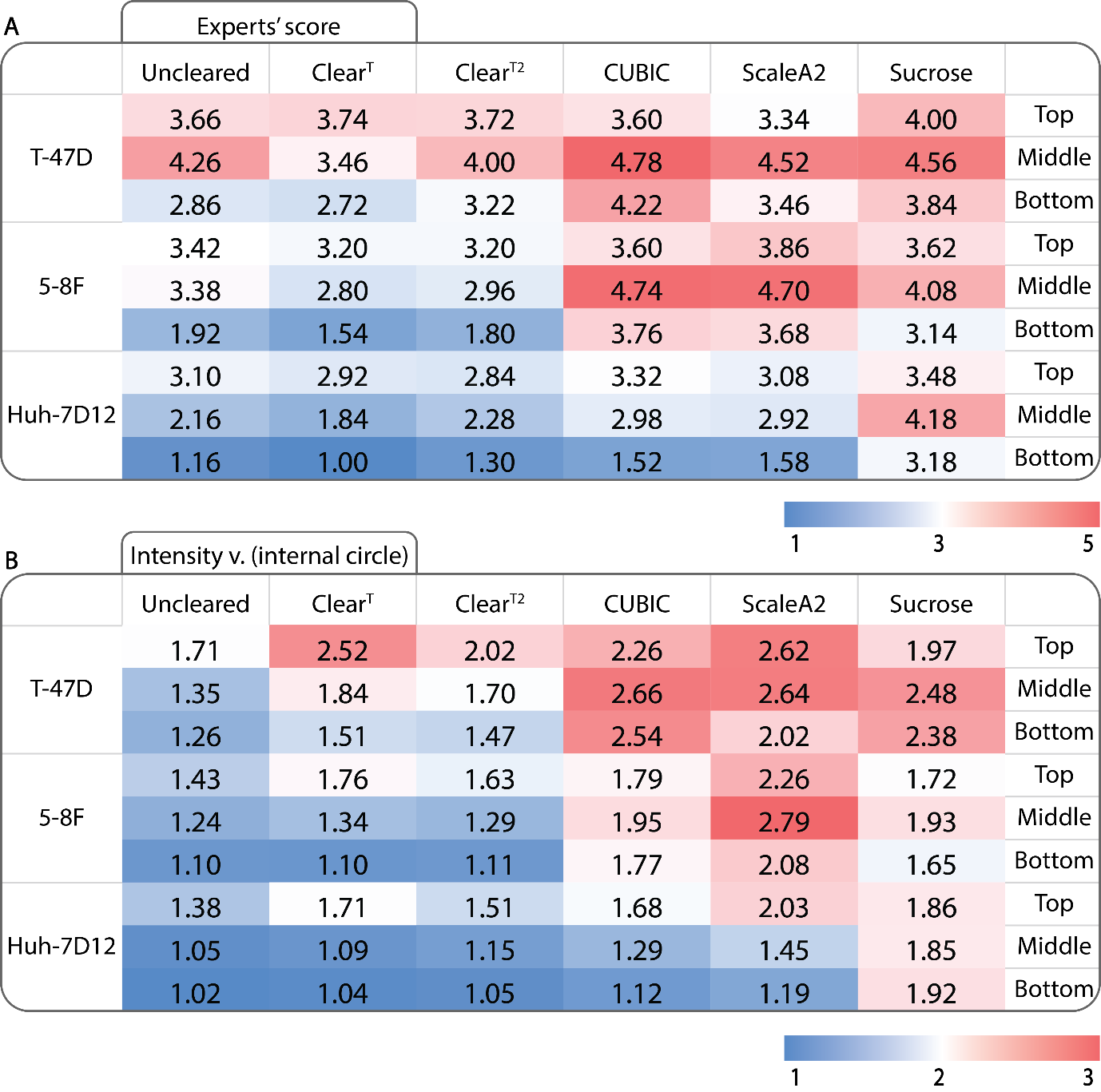
**

**Fig. S1 Heatmap representations of the dataset.**

(**A**) The average results for the ten experts’ assessment are visualized on a heatmap. The experts evaluated five spheroids from each group, but only one image from each region, and the average of their scores were represented. (**B**) For comparison, the quality assessment using intensity variance metric (with the internal circle option) was calculated based on the evaluation of five spheroids from each clearing protocol group. In this case the metric evaluated all the images from each region, and the average scores were visualized.

**
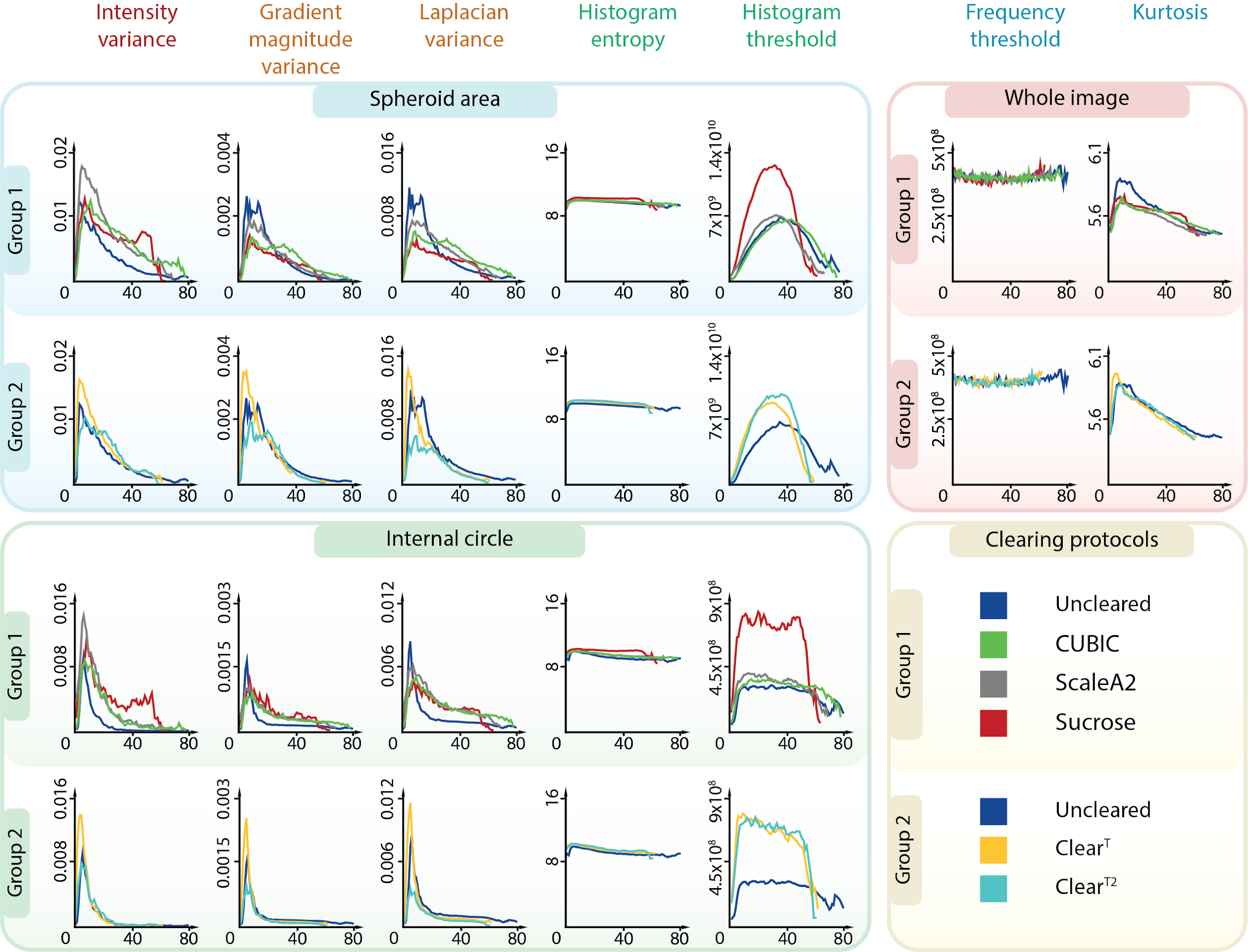
**

**Fig. S2** **Summary figure of the metrics on the Huh-7D12 spheroids.**

Huh-7D12 spheroids were evaluated with all the seven metrics tested. In case of intensity variance, gradient magnitude variance, Laplacian variance, histogram entropy, and histogram threshold metrics, the thresholded area of the spheroid and the internal circle options were used to assess transparency. Frequency threshold and kurtosis metrics assessed the whole image, with no option of internal evaluation. For a better visibility, the results for the optical clearing groups are separated into group 1 and group 2.


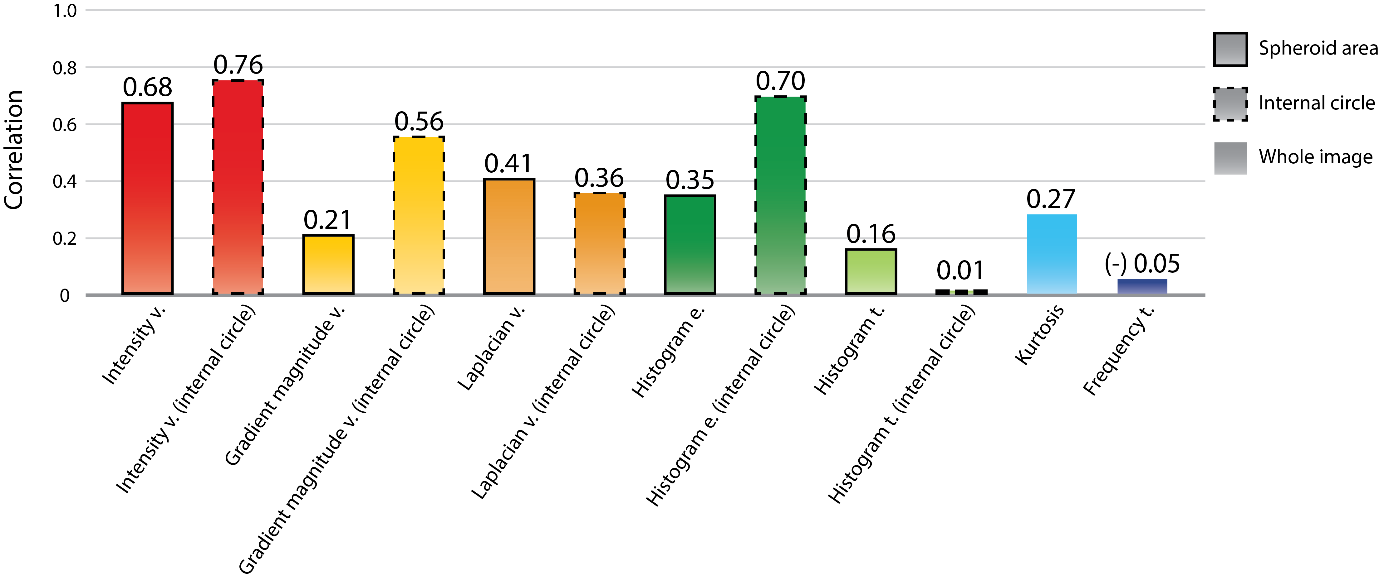


**Fig. S3** **Cell line-based correlation between metrics and experts’ evaluation.**

Results for the Pearson’s correlation analysis between the metrics and the experts’ assessment. Measurements obtained considering the cell lines individually. Bounding boxes with dashed lines represent the results of the internal circle assessment.

**
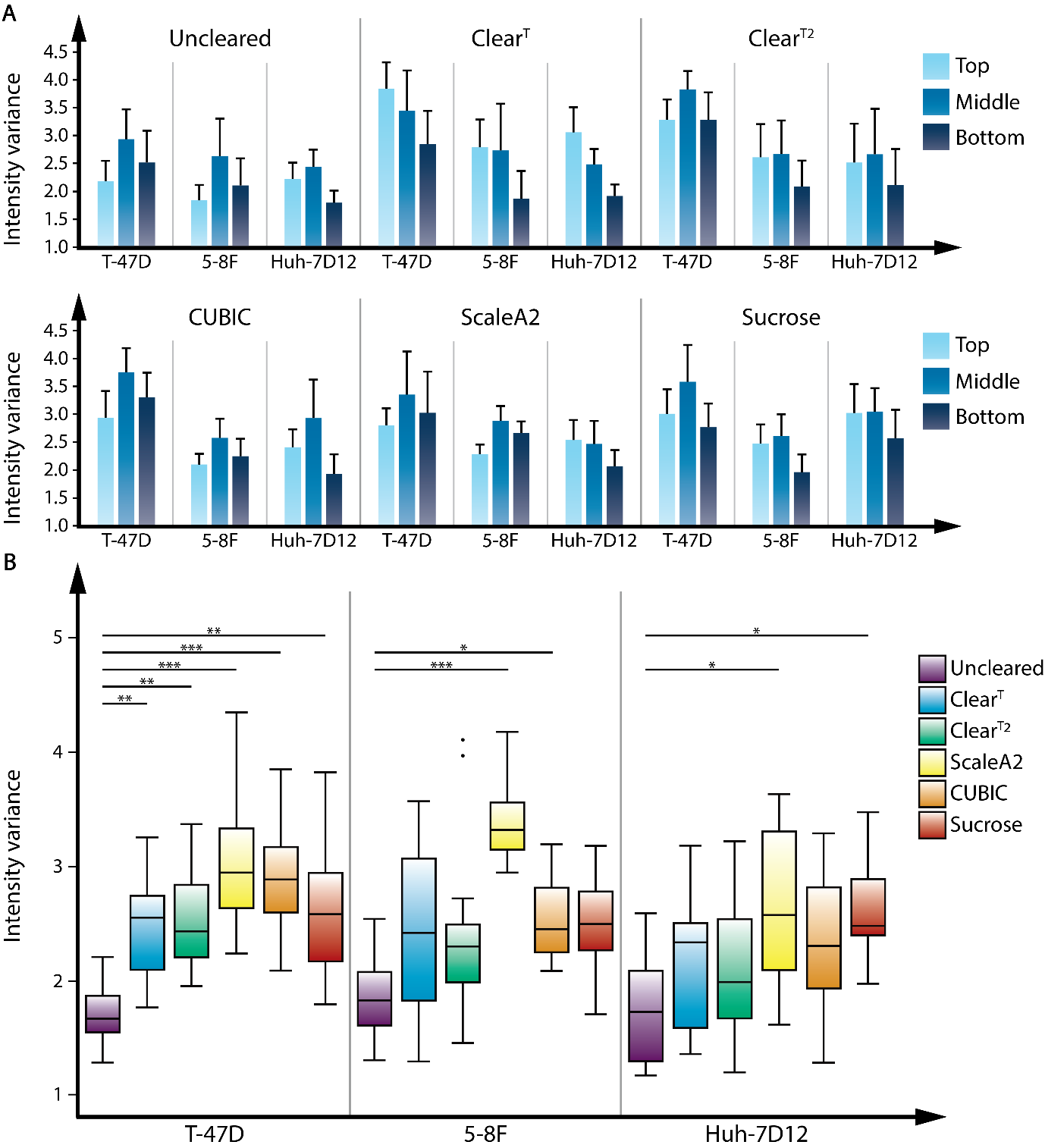
**

**Fig. S4** **Comparison of the effects of 5 optical clearing protocols on spheroids, using intensity variance metric on the whole spheroid.**

(**A**) Performance (efficacy) of the clearing protocols on the spheroid types derived from different cell lines. Three types of spheroids were compared at three regions (top, middle, bottom) using intensity variance metric, after applying each clearing protocol. The scores represent the results for the different types of spheroids treated with the same clearing protocol. (**B**) Comparison of the clearing protocols on each spheroid type to reveal the most appropriate method for each cell line. Intensity variance was used to assess the quality of each protocol. The efficacy of each clearing protocol was compared to the uncleared spheroids of the same type. Each cleared group contains 5 (top, middle and bottom), yielding 15 values per group for quality assessment. **p* ≤ 0.05; ***p* ≤ 0.01; ****p* ≤ 0.001.

**Supp. Note 1 Correlation results for all the metrics**

Here, we discuss the correlation of all the metrics. For the comparison, we computed the Pearson’s correlation coefficient between each metric and the experts’ evaluation used as the ground truth. For normalization, we used the highest value obtained considering the 90 spheroids all together. The correlation coefficients were higher in case of intensity variance, histogram entropy, and kurtosis metrics that resulted in 0.67, 0.49, and 0.54, respectively (**Fig. 4A**). Gradient magnitude variance, Laplacian, histogram threshold and frequency threshold metrics obtained a weak or no correlation (i.e. values < 0.5). Except for kurtosis and frequency threshold metrics which use the whole image only, for all the other metrics we also analysed the internal circle. The internal circle assessment improved the overall match with the experts’ evaluation and showed a higher correlation for intensity variance, gradient magnitude variance and histogram entropy metrics. The correlation coefficient for intensity variance and histogram entropy metrics improved from 0.67 to 0.80 and from 0.49 to 0.74, respectively (**Fig. 4A**). For gradient magnitude variance metric, it improved from 0.46 to 0.53. In all other cases, the internal circle assessment decreased the results: Laplacian variance from 0.03 to -0.01, and histogram threshold from 0.24 to 0.16. Next, we also normalized the cell lines individually (**Fig. S4**). The individual normalization decreased the correlation with almost every metric, except for the Laplacian metric, the intensity variance metric for the whole spheroid and the internal circle assessment of gradient magnitude variance. The correlation of the internal circle assessment resulted in 0.7 for histogram entropy and 0.76 for intensity variance, which accordingly showed the strongest correlation with the human scores either when applied to the whole spheroid or to the internal circle only.
